# An enduring legacy of discovery: Margaret Stirewalt

**DOI:** 10.1371/journal.pntd.0005714

**Published:** 2017-08-17

**Authors:** Lucie Henein, James J. Cody, Michael H. Hsieh

**Affiliations:** 1 Biomedical Research Institute, Rockville, Maryland, United States of America; 2 Division of Urology, Children’s National Health System, Washington, D.C., United States of America; 3 Department of Urology, The George Washington University, Washington, D.C., United States of America; University of Queensland, AUSTRALIA

Margaret A. Stirewalt, PhD (married Lincicome), known as “Peg” to friends and family, was a brilliant female scientist dedicated to the study of tropical medicine. Her seminal research centered on the study of schistosomiasis.

Dr. Stirewalt was born on 18 January 1911 in Hickory, North Carolina. She was the eldest of 4 children and her father was a farmer turned banker. She later moved to New Market, Virginia, where her grandfather was a Lutheran minister. These influences molded Dr. Stirewalt's spiritual faith and passion for science, education, and nature.

Dr. Stirewalt received her BA in 1931 at Randolph-Macon Woman’s College in Lynchburg, Virginia. She continued her studies, earning an MA at Columbia University, New York, in 1935 before going on to obtain a doctorate degree in 1938 from the University of Virginia. It is important to note that in the 1930s, it was still rare to find women who had achieved this level of education, particularly in the sciences. The fact that Dr. Stirewalt earned her PhD in a scientific field is laudable, but she did not stop there.

After receiving her PhD, Dr. Stirewalt joined the United States Navy during World War II, serving as an intelligence officer and commander. She then became a Naval Medical Officer at the Naval Medical Research Institute (NMRI) in Bethesda, Maryland. The 1940s were a challenging time for women to be successful scientists—or military officers, for that matter. With her strong-willed nature and intelligence, Dr. Stirewalt overcame these challenges and initiated a schistosomiasis research program. She authored over 50 peer-reviewed primary research papers, several of which have been cited more than 170 times. Her work on schistosome cercariae, the parasite stage that invades its definitive mammalian host, has been a particularly important contribution to the field of parasitology. She was one of the first scientists to clearly outline the process whereby these cercariae invade, through the use of remarkably detailed electron micrographs that illustrated these mechanisms (Fig 1).

**Fig 1:**
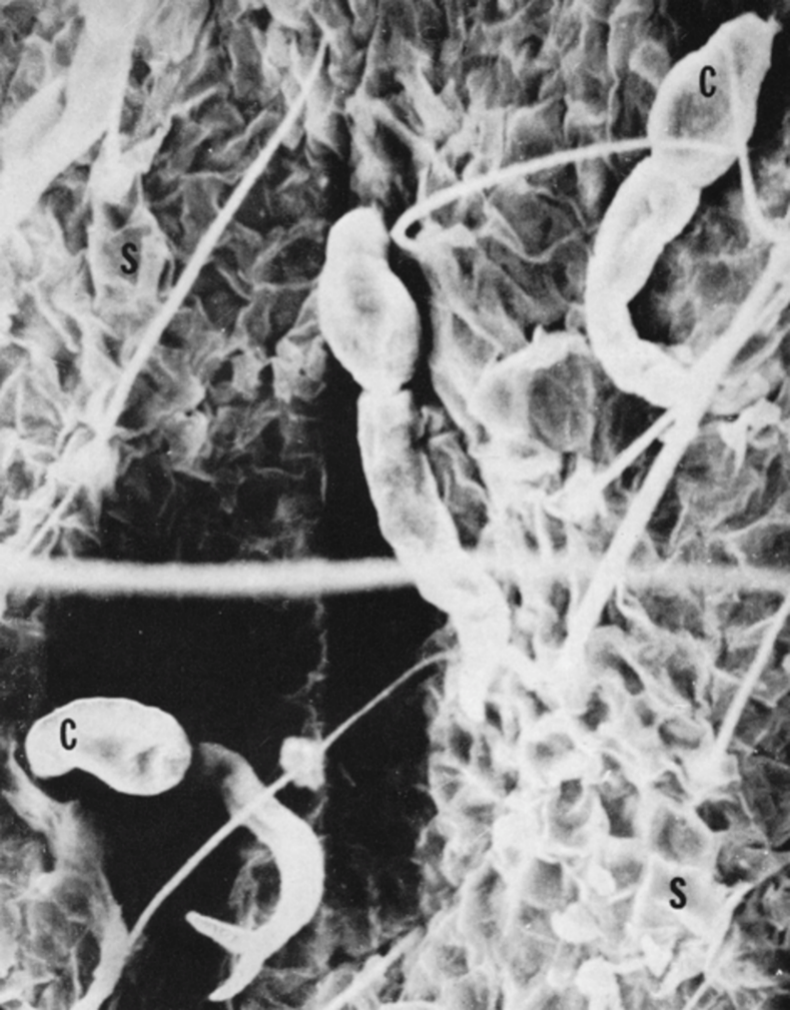
Electron micrograph of 3 schistosome cercariae (2 of which are marked with a “C”) in the process of invading the skin of a mouse ear. *Adapted from Stirewalt and Dorsey*, *Exp Parasitol*, *1974* [1].

Dr. Stirewalt maintained an active research program and published research articles regularly from the 1940s through the 1980s, with her last publication being a review article in 2002. Her initial work focused on infection of mammalian hosts by cercariae, including an early study on prevention of infection [2]. She was among the first to characterize the process of infection by cercariae and published a key study on skin penetration in five different hosts, including humans [3]. She also published a series of studies examining the serological responses to infection in multiple mammalian hosts [4–8]. As she continued her focus on cercariae, she was among the earliest to describe in detail the acetabular gland complex in *Schistosoma mansoni* and laid the groundwork for characterizing the secretory products of this complex [9]. Through the 1960s, she published numerous articles on the effects of various experimental conditions on the efficiency of cercarial infection of mice. Thorough studies such as these were critical in establishing many of the methods that continue to be used for propagating *Schistosoma* sp. in the laboratory, such as infection by cercariae and collection of schistosomules. Through the 1970s and into the 1980s, she continued publishing on the infection process, including studies of the secretion of enzymes by cercariae and the histology of infected host tissue. Also in the 1980s, she published several studies on the cryopreservation of schistosomules, paving the way for some of the earliest vaccine studies. For example, she demonstrated that vaccination of challenged baboons by cryopreserved, irradiated schistosomules led to a reduction in granuloma size [10]. Her later work focused on such vaccine studies as well as methods for generating schistosomules in the laboratory.

Following her departure from the NMRI, Dr. Stirewalt established a schistosomiasis lab at the Biomedical Research Institute (BRI), currently located in Rockville, Maryland. In doing so, she ensured the continuation of her groundbreaking research and started a schistosomiasis resource center to provide researchers around the world with samples of the *Schistosoma* parasite at various stages in its life cycle. Dr. Stirewalt was also a pioneer for women serving as active and influential members of the scientific community, given that she received multiple awards in research and service, served on grand review committees, and was a member of the editorial boards of numerous scientific journals.

Dr. Stirewalt’s extraordinary achievements in the world of academia bring to light her love of learning. She believed that education was the most powerful tool that could be passed down through generations to heal the environment and battle disease and human poverty. She supported her younger brother financially through medical school and his specialization in gerontology. Dr. Stirewalt also served as a well-respected teacher and mentor to a number of contemporary parasitologists, such as Dr. Fred Lewis, formerly of the BRI, and Dr. Dan Colley of the University of Georgia. Dr. Carolyn Cousin, her final postdoctoral fellow, said of Dr. Stirewalt that “the treasure that she truly was is impossible to capture” and called her “outstanding as a scientist and a human being.” In order to ensure the continuing advancement of research and teaching in the field of parasitology, a professorship was established with her husband Dr. David Lincicome. Together, they created the Harley Jones Van Cleave Professorship in Life Sciences at the University of Illinois at Urbana–Champaign.

Interestingly, Dr. Stirewalt also established a professorship at the Virginia Polytechnic Institute to support education and research on goats, based on her conviction that goats would replace the cow in the agricultural world. While seemingly tangential to the main focus of her academic efforts, the reason for this professorship is easily explained by her interests outside of the laboratory. After retiring from a long and prolific career in 1983, Dr. Stirewalt spent time cultivating her outside interests such as her love of goats, dogs, and sheep (Fig 2).

**Fig 2:**
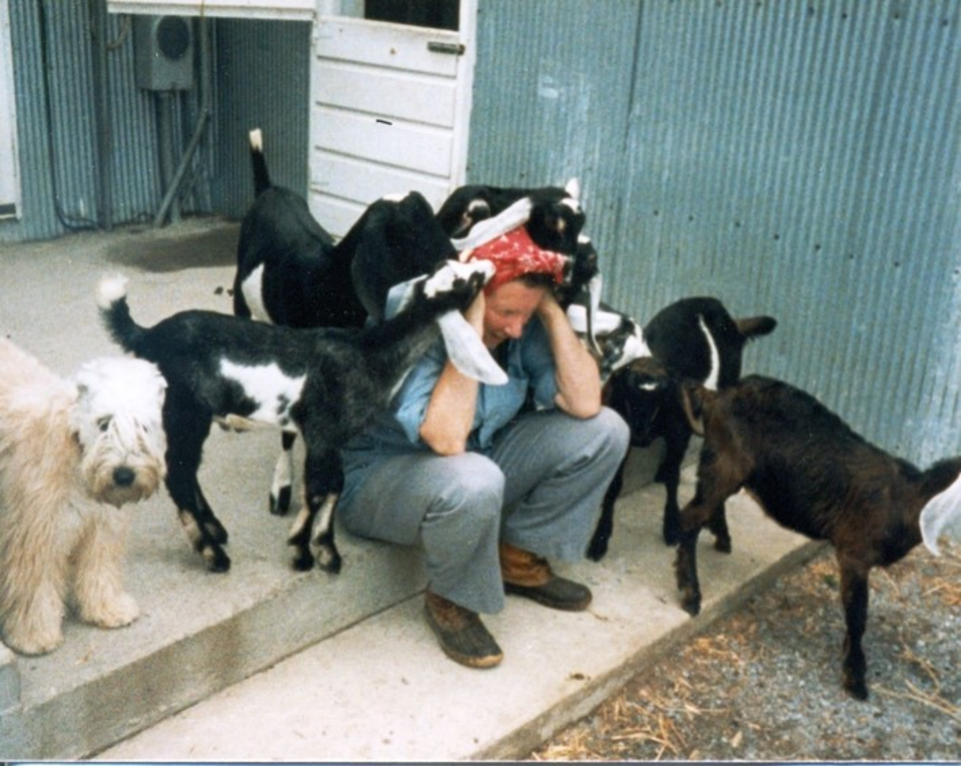
Peg Stirewalt with some Nubian goats and one of her Wheaten terriers.

A member of the Virginia Dairy Goat Association, she acquired extensive experience in milking Nubian goats. However, according to her husband, Dr. Stirewalt would occasionally have to deal with a certain temperamental doe that would squat down on the milking stand when she walked into the room. She particularly enjoyed caring for the baby goats. Dr. Stirewalt also had a passion for nature. She was an avid observer of birds, flowers, and trees. Throughout her life, she spent countless hours hiking and maintaining parts of the Appalachian Trail (Fig 3), and during her retirement, she proudly built upon her efforts to educate others about the wonders of the outdoors. Dr. Stirewalt passed away in 2003, and just as she left behind a towpath in the trails she hiked, she left behind an enduring legacy for male and female scientists alike. Her pathways to success are an example for us all.

**Fig 3:**
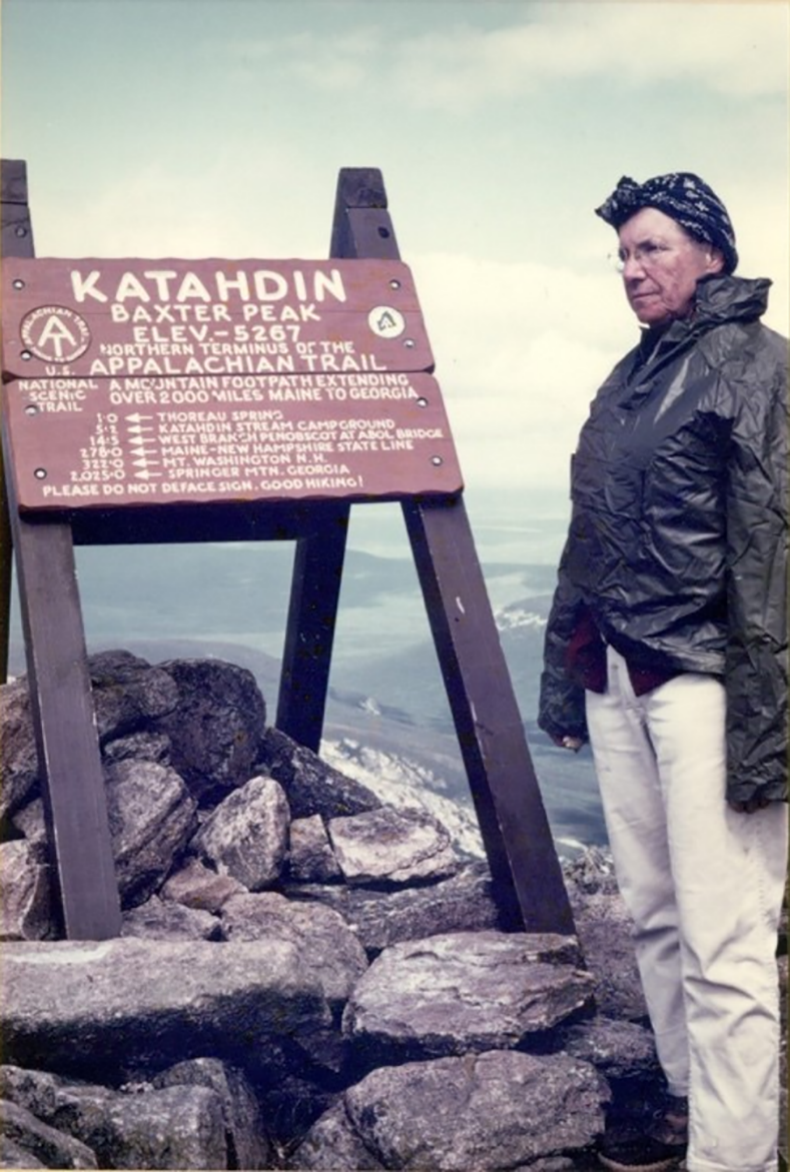
Peg Stirewalt in later years, on Mt. Katahdin, the northern terminus of the Appalachian Trail.

To honor this legacy, the BRI established the Margaret A. Stirewalt Endowed Directorship in 2014. The goal of this endowed position was to recruit an accomplished schistosomiasis researcher to the BRI in order to help continue and expand upon its tradition of scientific leadership in the field. An ancillary objective of the directorship was to provide a crucial measure of stability for schistosomiasis research in this era of limited research funding. Dr. Michael Hsieh was selected as the inaugural recipient of the Stirewalt endowment. Since its recent inception, the endowment has already enabled the training of 3 postdoctoral fellows, 2 master’s students, 1 college student, and 2 high school students. The Stirewalt endowment has also facilitated additional NIH and industry funding for the BRI to conduct schistosomiasis research. Finally, the endowment has funded studies resulting in 4 publications [11–14]. Thus, the Margaret A. Stirewalt Endowed Directorship has been a successful commemoration of one of the BRI's founding scientists, and we anticipate that it will continue to be for years to come.

## Supporting information

S1 Fig “Unheralded—An Ode to My Mentor.” This original poem by Dr. Carolyn Cousin was written to celebrate the enduring legacy of Dr. Stirewalt as a scientist and mentor. Dr. Cousin was the last postdoctoral fellow to be trained by Margaret Stirewalt and maintained a professional association with her for years thereafter. “Unheralded” was originally delivered at a memorial service for Dr. Stirewalt and was donated by the author.
(PDF)
