# Supplementary material for: An enduring legacy of discovery: Margaret Stirewalt
Source: PLoS Negl Trop Dis. 2017 Aug 17;11(8):e0005714. doi: 10.1371/journal.pntd.0005714 (PMC5560522; doi:10.1371/journal.pntd.0005714)
Supplement: S1 Fig — This original poem by Dr. Carolyn Cousin was written to celebrate the enduring legacy of Dr. Stirewalt as a scientist and mentor. Dr. Cousin was the last postdoctoral fellow to be trained by Margaret Stirewalt and maintained a professional association with her for years thereafter. “Unheralded” was originally delivered at a memorial service for Dr. Stirewalt and was donated by the author. (PDF) [file pntd.0005714.s001.pdf]

UNHERALDED
An ode to my mentor

Move her to the head of the line
Not because she asked for it,
But because she earned it,
I stand to herald her praises

So many times she rose to the occasion
With skillful hands and little demand
Often taking the sole stand
While others erected barriers in the sand.

She never asked for your praises
When they came, she would often walk away
She never sought your adoration
Her deeds came from love and dedication.

My mentor was the lead
Not because she asked for it
But because she earned it
I wished all could know her deeds

May her legacy live on until all can see
How her actions elevated many, even the excluded like me.
She taught me to stand tall,
Leap over barriers and high walls,
And to focus my sights toward the sky
And with her propelling skills taught me how to fly

Like Plato's students, I learned at her knee
And it did not take me long to see,
That not only did she break the ground for me,
But for a whole generation, yet to be.

C. E. Cousin

Carolyn Cousin, Ph.D.
Professor
Division of Science and Mathematics
University of the District of Columbia
4200 Connecticut Ave, NW
Building 44, Room 200-07
Washington, DC 20008
Tel: (202) 274-5874
Fax: (202) 274-5773
E-mail: ccousin@udc.edu
